# Prolonged Prophylactic Protection from Botulism with a Single Adenovirus Treatment Promoting Serum Expression of a VHH-Based Antitoxin Protein

**DOI:** 10.1371/journal.pone.0106422

**Published:** 2014-08-29

**Authors:** Jean Mukherjee, Igor Dmitriev, Michelle Debatis, Jacqueline M. Tremblay, Gillian Beamer, Elena A. Kashentseva, David T. Curiel, Charles B. Shoemaker

**Affiliations:** 1 Department of Infectious Disease and Global Health, Tufts Cummings School of Veterinary Medicine, North Grafton, Massachusetts, United States of America; 2 Department of Radiation Oncology, Washington University, St. Louis, Missouri, United States of America; CEA (Atomic and alternative energies commission), France

## Abstract

Current therapies for most acute toxin exposures are limited to administration of polyclonal antitoxin serum. We have shown that VHH-based neutralizing agents (VNAs) consisting of two or more linked, toxin-neutralizing heavy-chain-only V_H_ domains (VHHs), each binding distinct epitopes, can potently protect animals from lethality in several intoxication models including Botulinum neurotoxin serotype A1 (BoNT/A1). Appending a 14 amino acid albumin binding peptide (ABP) to an anti-BoNT/A1 heterodimeric VNA (H7/B5) substantially improved serum stability and resulted in an effective VNA serum half-life of 1 to 2 days. A recombinant, replication-incompetent, adenoviral vector (Ad/VNA-BoNTA) was engineered that induces secretion of biologically active VNA, H7/B5/ABP (VNA-BoNTA), from transduced cells. Mice administered a single dose of Ad/VNA-BoNTA, or a different Ad/VNA, via different administration routes led to a wide range of VNA serum levels measured four days later; generally intravenous > intraperitoneal > intramuscular > subcutaneous. Ad/VNA-BoNTA treated mice were 100% protected from 10 LD_50_ of BoNT/A1 for more than six weeks and protection positively correlated with serum levels of VNA-BoNTA exceeding about 5 ng/ml. Some mice developed antibodies that inhibited VNA binding to target but these mice displayed no evidence of kidney damage due to deposition of immune complexes. Mice were also successfully protected from 10 LD_50_ BoNT/A1 when Ad/VNA-BoNTA was administered up to 1.5 hours post-intoxication, demonstrating rapid appearance of the protective VNA in serum following treatment. Genetic delivery of VNAs promises to be an effective method of providing prophylactic protection and/or acute treatments for many toxin-mediated diseases.

## Introduction

Botulism is a flaccid paralysis caused by exposure to Botulinum neurotoxin (BoNT) that results primarily from ingestion of contaminated foods, although the risk of exposure through deliberate events is considered sufficiently high to list BoNT as a Category A Priority Pathogen. Toxin exposure is commonly treated by administration of antitoxin serum, generally prepared from large animals immunized with inactivated toxin [1]–[3]. Such antiserum products possess safety risks and are difficult to develop, produce and maintain. Antiserum is also not practical for prophylactic protection of people that are considered at-risk of toxin exposures. BoNT antiserum alternatives, such as monoclonal antibodies (mAbs) are under development [4] and other strategies are in the research stage [1].

We have reported the use of an alternative antitoxin strategy [5] which employs ‘VHH-based neutralizing agents’ (VNAs) consisting of linked 14 kDa camelid heavy-chain-only V_H_ domains (VHHs), produced as heteromultimers, that bind and neutralize toxin targets. VNAs were found to be much more potent at neutralization of Shiga toxins (Stx1, Stx2) [6] and ricin [7] than pools of the component VHHs. The VNAs also contain several copies of an epitopic tag recognized by an anti-tag mAb. Co-administration of the anti-tag mAb, called the effector Ab (efAb), enhances therapeutic efficacy in some toxin models [5]–[7], probably by promoting toxin clearance through the liver [8].

VNA antitoxins offer the potential for genetic delivery using vehicles that lead to patient expression of antitoxin protein. Ideally, such a vehicle would permit single-treatment, prophylactic, long-term protection from toxins. A wide variety of genetic delivery vehicles have already been developed including direct administration of DNA and RNA, recombinant adenoviruses (Ad) [9]–[13], and adeno-associated virus (AAV) [14]–[17]. Furthermore, gene delivery vehicles can effectively promote in vivo expression of a range of antibody species for passive immunotherapy [9]–[11], [18].

In this paper, we report the use of a recombinant, replication-incompetent, human Ad serotype 5 (Ad5) vector that promotes de novo secretion of antitoxin VNAs into serum. Ad5 was selected as the VNA delivery vehicle due to its ability to promote rapid de novo expression of recombinant products following treatment, its wide use both as a research tool and a therapeutic agent in the clinic, and the fact that vector DNA does not integrate into host cells. The Ad/VNA-BoNTA vector we generated produces a potent BoNT/A1 antitoxin VNA (H7/B5) that we previously described [5]. Here, the beneficial effects of H7/B5 are improved by engineering an albumin-binding peptide (ABP) that enhances serum persistence. We demonstrate that a single treatment with Ad/VNA-BoNTA protects mice for several months from subsequent BoNT/A1 challenge, yet acts sufficiently rapid to be effective when administered shortly after toxin exposure.

## Results

### Prolonging serum persistence of antitoxin VNAs

Recombinant antibody agents such as VHHs have short serum half-lives [19] which we sought to improve by appending a 14 amino acid murine albumin-binding-peptide (ABP), DICLPRWGCLEWED [20] to the the carboxyl-terminus of H7/B5. The resulting VNA was expressed as a recombinant thioredoxin (Trx) fusion protein, Trx/H7/B5/ABP, and purified. ABP in the VNA dramatically improved affinity for mouse serum and variably improved VNA affinity to other mammalian sera (Figure S1).

**Figure 1:**
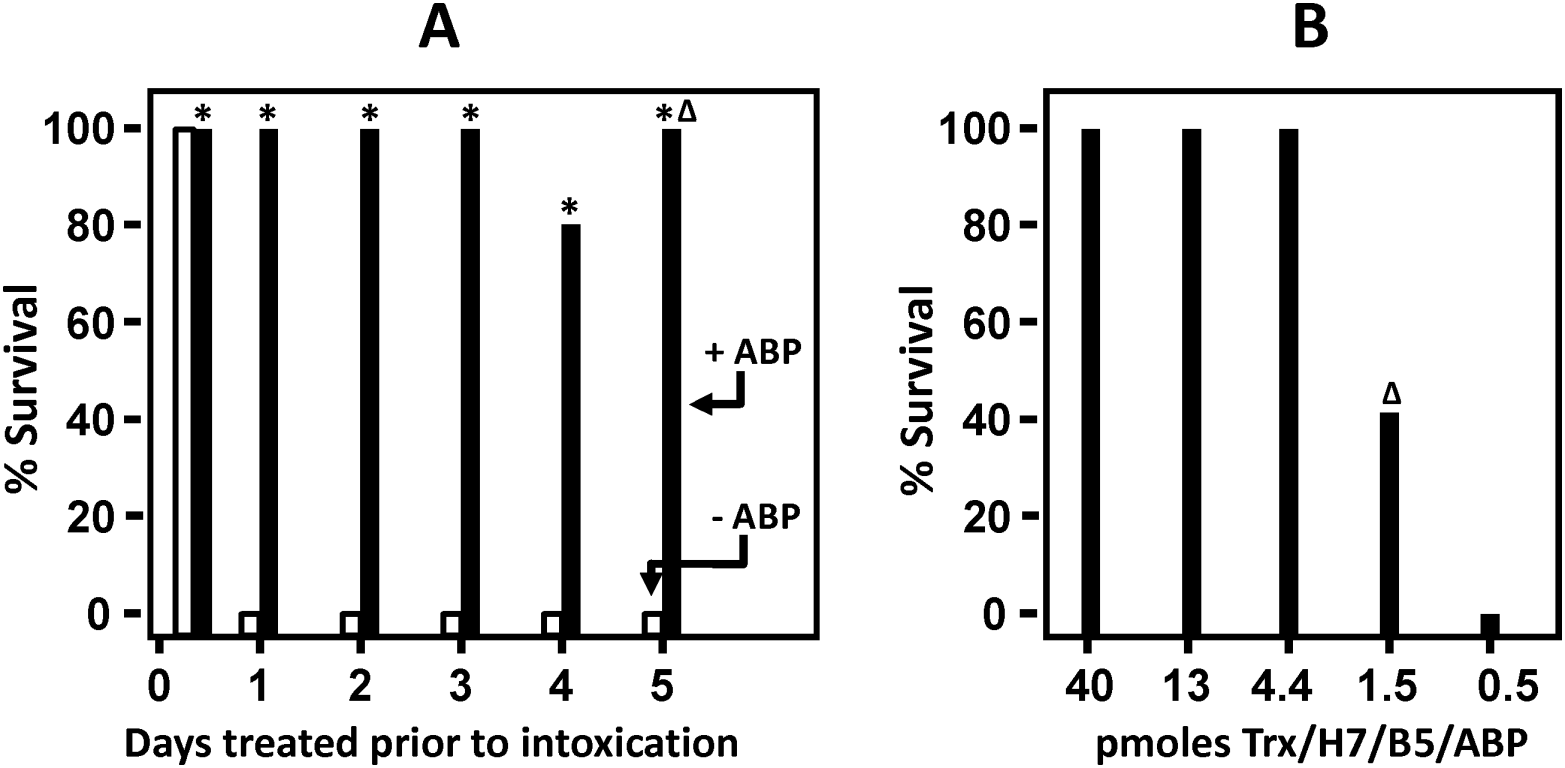
Albumin binding peptide (ABP) extends the serum persistence of VNA bioactivity. (**A**) Protection from BoNT/A1 in mice treated with Trx/H7/B5 or Trx/H7/B5/ABP at various times prior to intoxication. Groups of five mice each (60 mice total) were treated with a known protective dose of 2 µg (40 pm) VNA and later challenged with 10 LD_50_ of BoNT/A1 at the indicated times post-treatment. Bars indicate the % survival: Trx/H7/B5, white bar; Trx/H7/B5/ABP, black bar. Groups with no survival show a short bar ending at 0. A delta (Δ) indicates that all mice in this group displayed discernable abdominal breathing symptoms of botulism. An asterisk indicates a significant difference (p<0.05) from the control group. Results reported are representative of three similar studies. (**B**) Minimum dose of Trx/H7/B5/ABP that protects from 10 LD_50_ BoNT/A1. Groups of five mice (25 mice total) were treated with the indicated dose of Trx/H7/B5/ABP and then challenged 6 hours later with 10 LD_50_ BoNT/A1. Black bars indicate % survival and a delta (Δ) indicates that surviving mice displayed severe abdominal breathing symptoms of botulism. An asterisk indicates a significant difference (p<0.05) from the control group. Results reported are representative of three similar studies.

To determine whether the ABP extends the effective serum half-life of the VNA in vivo, 2 µg Trx/H7/B5 or Trx/H7/B5/ABP were administered to mice at various times prior to intoxication with 10 LD_50_ of BoNT/A1. As shown in Figure 1A, groups of five mice were mostly protected (14/15 in three studies) from lethality by treatment 6 hours earlier with Trx/H7/B5, although some mice (5/15 in three studies) showed mild abdominal breathing symptoms of botulism. No mice survived the toxin challenge when Trx/H7/B5 was administered one day earlier, and time to death in this group was not significantly different from controls (not shown). Control mice receiving no treatment all died within 24 hours of intoxication (not shown). In contrast, mice given 2 µg Trx/H7/B5/ABP all survived toxin challenge administered 1, 2, 3, or 4 days later and displayed no symptoms of botulism. Mice intoxicated 5 days after treatment with Trx/H7/B5/ABP survived and remained active, but all (5/5) displayed severe abdominal breathing symptoms of botulism. Thus ABP extended the effective serum life of a 2 µg dose of Trx/H7/B5 by about 5 days.

To estimate the serum half-life (T_1/2_) of functional Trx/H7/B5/ABP, we first assessed the ‘minimum effective dose’. This was performed by treating mice with a range of doses and then challenging with 10 LD_50_ of BoNT/A1 the same day. As shown in Figure 1B, under these conditions, the minimum effective dose of Trx/H7/B5/ABP was about 4 pmoles or 0.2 µg (Figure 1B). Based on this, mice pre-treated with 40 pmoles (2 µg) of Trx/H7/B5/ABP would not be expected to survive when the VNA agent has undergone more than three T_1/2_. Thus, the observation that Trx/H7/B5 protected mice for about 6 hours (Figure 1A) indicates the effective T_1/2_ is 1–2 hours. Trx/H7/B5/ABP protected mice for about 5 days, indicating the effective T_1/2_ is 1–2 days.

### Serum VNA levels in mice following Ad/VNA transduction

An Ad5-based vector, Ad/VNA-BoNTA, was constructed in which DNA encoding H7/B5/ABP (VNA-BoNTA) was fused to a mammalian signal peptide (transcription unit diagram Figure S2A) to promote secretion of VNA-BoNTA (aa sequence Figure S2B) from transduced cells. To test Ad serum expression of a second VNA, another vector, Ad/VNA-Stx, was constructed to express a VHH heterotrimer (A9/A5/G1 [6]) recognizing both Shiga toxins Stx1 and Stx2 (VNA-Stx, aa sequence Figure S2C). The VNAs in both vectors contained ABP at the carboxyl terminus. Mammalian cells, HeLa and A549, transduced by 10^4^ virus particles (vp) of Ad/VNA-BoNTA per cell efficiently secreted VNA-H7/B5/ABP into the medium for several days, achieving VNA levels of about ∼10 µg/ml. Similar results were obtained with Ad/VNA-Stx, although VNA levels in medium were ∼1 µg/ml. Transducing cells with a 10 fold lower dose of either virus led to proportionately reduced VNA levels in the medium (data not shown).

The two replication-incompetent Ad/VNA vectors were then tested for the ability to promote serum expression of functional VNA following virus administration in mice. Initially groups of five mice each were given 3×10^10^ vp of Ad/VNA-BoNTA or Ad/VNA-Stx via four different routes and bled at euthanasia four days later. Serum VNA levels were quantified using toxin-capture dilution ELISAs compared with normal mouse serum containing known amounts of each VNA (Figure S3). As shown in Tables 1 and 2, intravenous (iv) administration resulted in higher levels of serum VNA with both Ad/VNA vectors when compared to intraperitoneal (ip), intramuscular (im) or subcutaneous (sq) routes. VNA levels ranged widely in all groups although, surprisingly, most mice achieved 100 µg/ml or higher serum VNA levels four days following iv administration.

**Table 1:** Serum VNA-BoNTA levels following Ad/VNA-BoNTA administration via different routes.

Route^a^	Estimated serum VNA-BoNTA levels (nM)^b^					Average(nM)	Average(µg/ml)
	Mouse 1	Mouse 2	Mouse 3	Mouse 4	Mouse 5		
iv	6000	30000	50000	10000	50000	29000	970
ip	0.015	4000	4000	0.03	1000	1800	60
im	ND	1000	1000	2000	800	960	32
sq	400	100	1000	200	40	350	12

ND- not determined.

a Routes by which Ad was administered; iv-intravenous, ip-intraperitoneal, im-intramuscular, sq-subcutaneous.

b Serum was collected 4 days post-treatment and VNA levels estimated by dilution ELISA for binding to toxin targets.

**Table 2:** Serum VNA-Stx levels following Ad/VNA-Stx administration via different routes.

Route^a^	Estimated serum VNA-Stx levels (nM)^b^					Average(nM)	Average(µg/ml)
	Mouse 1	Mouse 2	Mouse 3	Mouse 4	Mouse 5		
iv	6000	400	4000	2000	6000	3700	185
ip	600	800	200	800	400	560	28
im	80	60	160	80	40	84	4
sq	6	20	4	1	20	10	0.5

a Routes by which Ad was administered; iv-intravenous, ip-intraperitoneal, im-intramuscular, sq-subcutaneous.

b Serum was collected 4 days post-treatment and VNA levels estimated by dilution ELISA for binding to toxin targets.

### Ad/VNA-BoNTA provides long term protection from BoNT/A1 challenge

To assess the duration of protection from BoNT/A1 intoxication by a single administration of Ad/VNA-BoNTA, two long-term studies were performed. In both studies, groups of five mice each were injected iv with 3×10^10^ vp of Ad/VNA-BoNTA or a control virus promoting expression of an irrelevant VHH heterodimer VNA (Ad/VNA-control) having no detectable binding to BoNT/A1. At various times following treatment, one treatment group and one control group of mice were challenged with 10 LD_50_ of BoNT/A1 and monitored for symptoms and survival.

**Figure 2:**
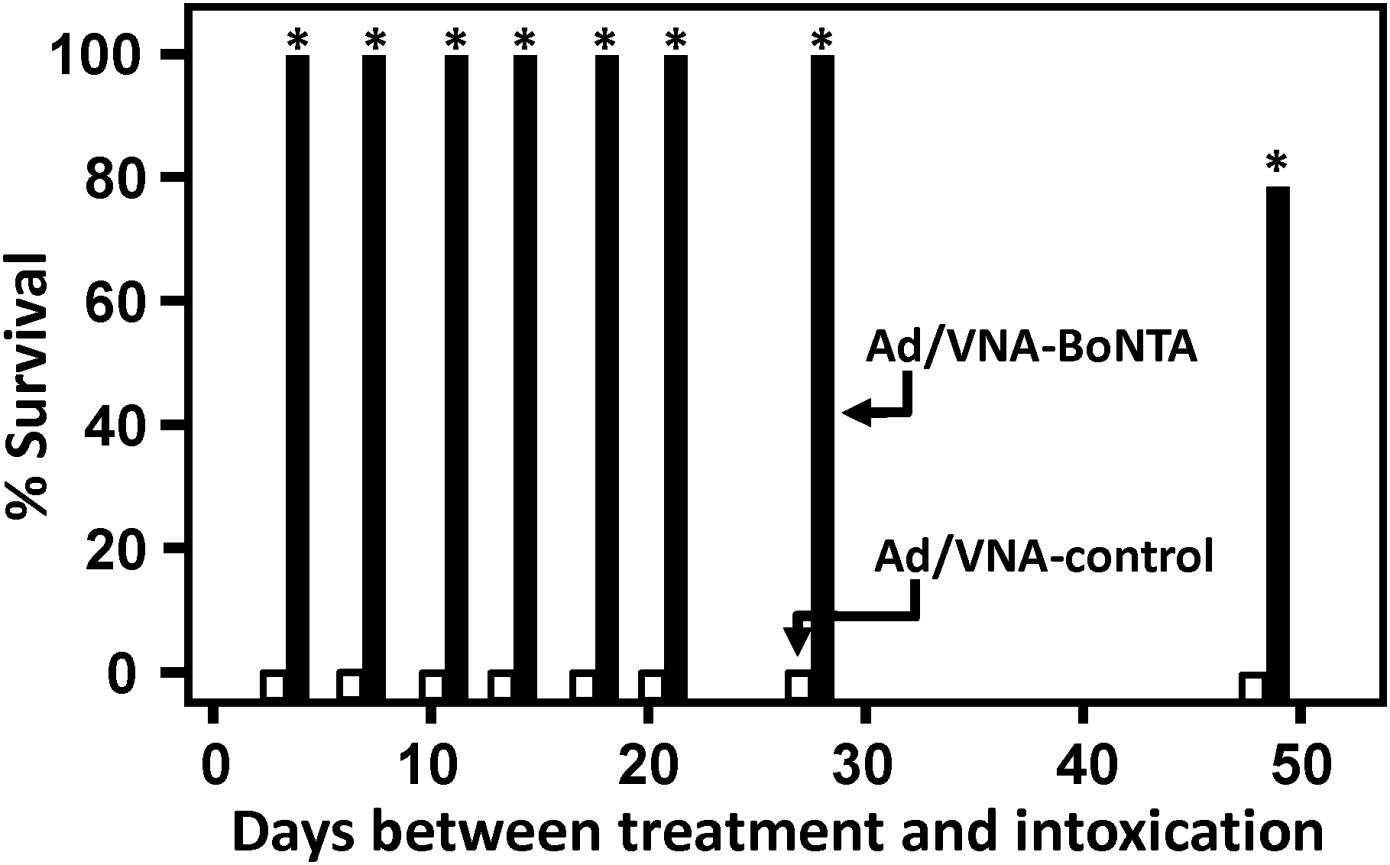
Ad/VNA-BoNTA treatment provides long term protection from botulism in mice- study 1. Groups of five mice (80 mice in total) were treated with 3×10^10^ vp of Ad/VNA-BoNTA or Ad/VNA-control on day 0 and one group treated with each of the two Ad/VNAs were administered 10 LD_50_ of BoNT/A1 at the various times post-treatment. Black bars indicate % survival of mice treated with Ad/VNA-BoNTA and later intoxicated at the indicated times. White bars indicate % survival of mice treated with Ad/VNA-control. Groups with no survival show a short bar ending at 0. An asterisk indicates a significant difference (p<0.05) from the control group.

**Figure 3:**
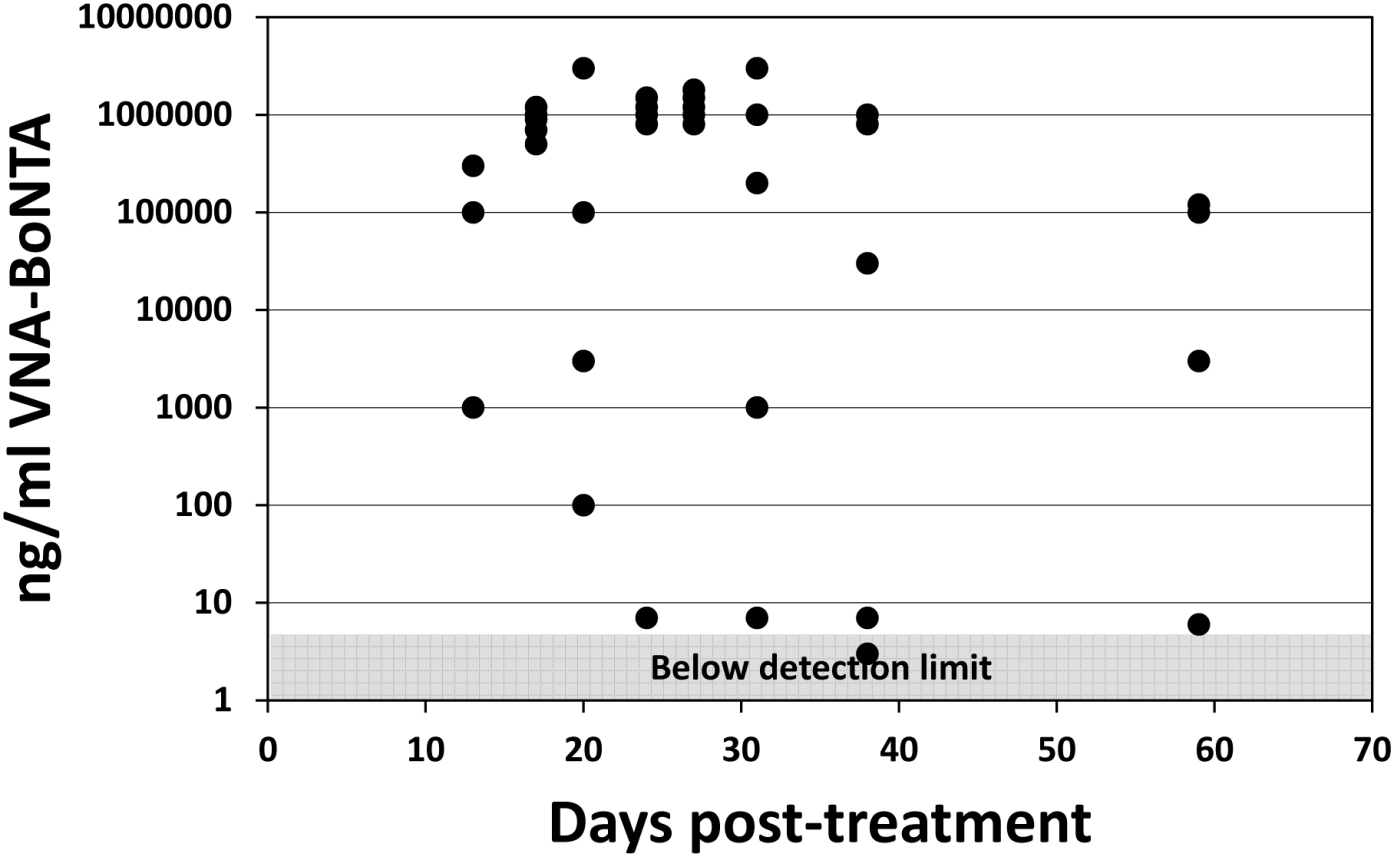
Serum levels of VNA-H7/B5/ABP following Ad/VNA-BoNTA treatments- study 1. All mice that survived BoNT/A1 treatments in study 1 were bled 10–11 days following intoxication. Serum from these terminal bleeds was assessed for serum VNA-BoNTA levels as described in Methods and Materials. The VNA concentration (ng/ml) from each individual mouse serum is represented as a black dot shown at the indicated time of collection, post-treatment. Dots within the shaded area all represent mice with VNA levels below the level at which the signals are at least two-fold over background and cannot be confidently distinguished from serum from control mice.

In study 1, numerous groups of mice were treated with Ad/VNA-BoNTA or Ad/VNA-control and one group of mice treated with each Ad vector were challenged with toxin at different times during the subsequent 7 weeks (Figure 2). As expected, all groups of mice pre-treated with the Ad/VNA-control died within a day following intoxication with BoNT/A1. All seven groups of mice pre-treated with Ad/VNA-BoNTA, and subsequently challenged within 4 weeks, survived toxin challenge and displayed no symptoms of botulism. In the final group of mice, challenged 7 weeks after pre-treatment with Ad/VNA-BoNTA, one of five mice failed to survive toxin challenge while the other four mice displayed no symptoms. Sera from all mice that survived 1 week post 10 LD_50_ challenge with BoNT/A1 were assayed for VNA-BoNTA levels (Figure 3). Serum VNA levels ranged widely from below detection levels to 3 mg/ml. Interestingly, the VNA-BoNTA level in one mouse that survived challenge at four weeks post-treatment was below ∼5 ng/ml, yet this was apparently sufficient to fully protect the mouse from botulism.

**Figure 4:**
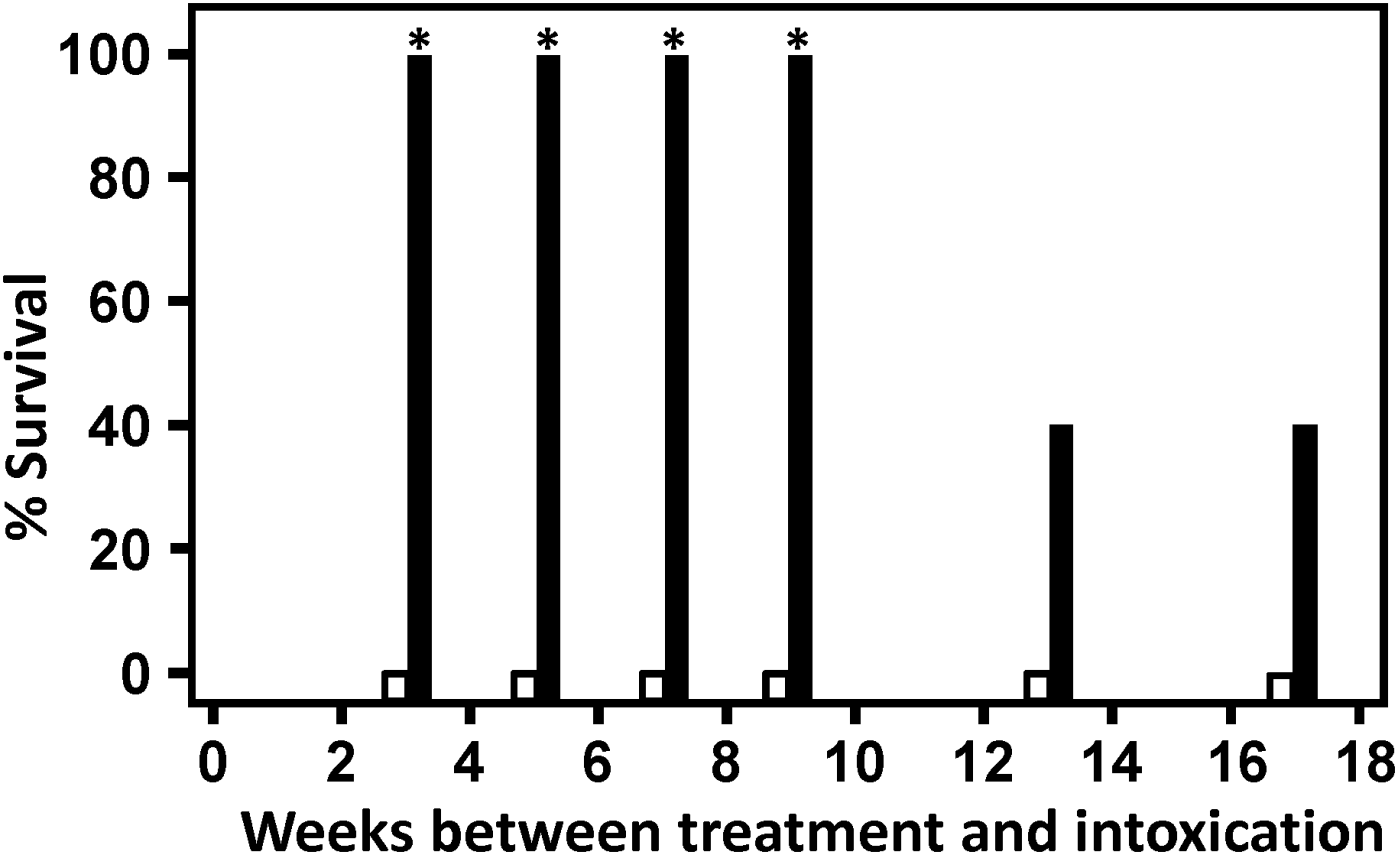
Ad/VNA-BoNTA treatment provides long term protection from botulism in mice- study 2. Groups of five mice (60 mice in total) were treated and challenged as for study 1 (Figure 2) although the timeframe was extended to four months. Black bars indicate % survival of mice treated with Ad/VNA-BoNTA and later intoxicated at the indicated times. White bars indicate % survival of mice treated with Ad/VNA-control. Groups with no survival show a short bar ending at 0. An asterisk indicates a significant difference (p<0.05) from the control group.

**Figure 5:**
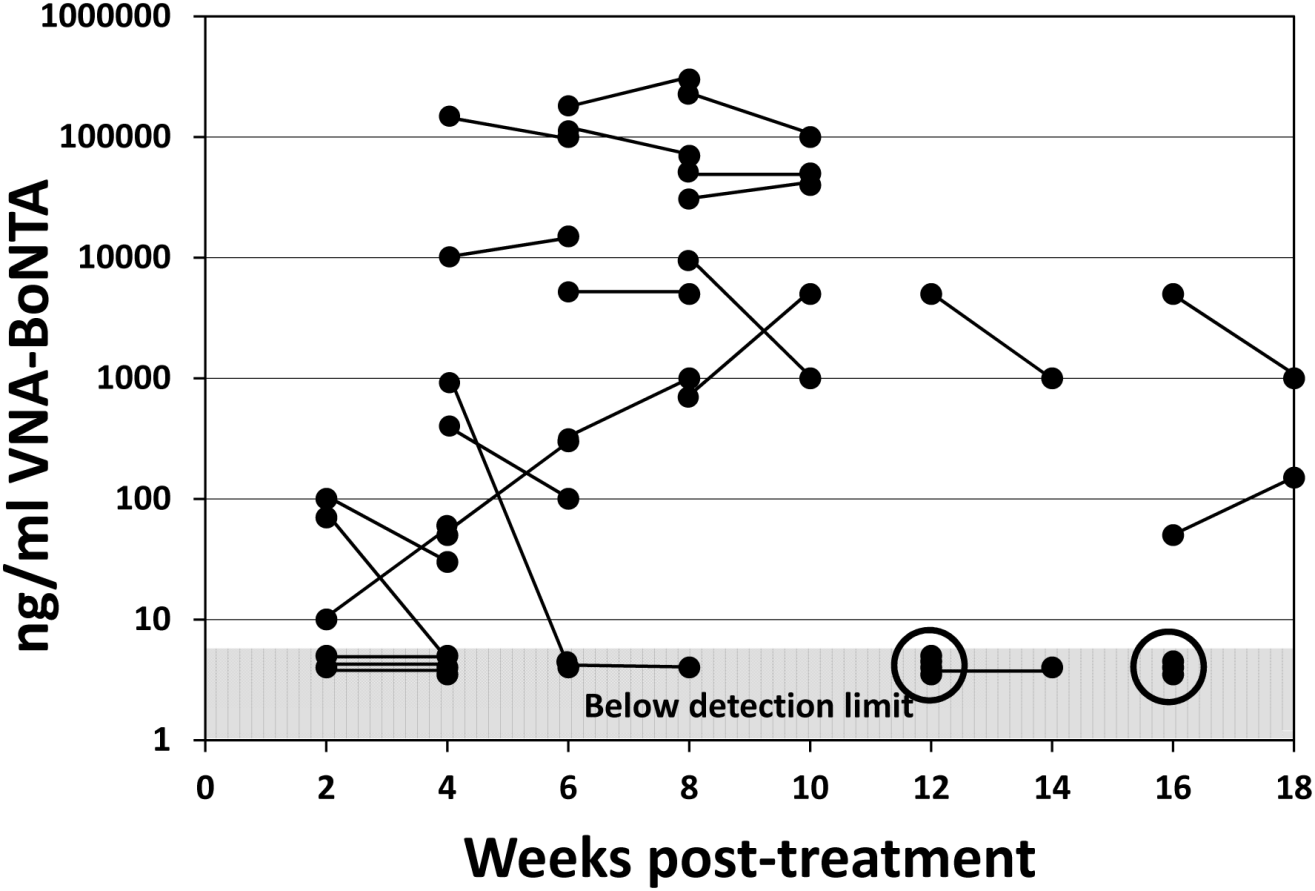
Serum levels of VNA-BoNTA following Ad/VNA-BoNTA treatments- study 2. Mice were treated with Ad/VNA-BoNTA and intoxicated as in study 1. Here, mice were bled one week prior to intoxication and survivors were also bled one week post-intoxication. VNA-BoNTA serum levels were quantified as described in Methods and Materials. The VNA concentration (ng/ml) from a single mouse serum is represented as a black dot. Lines connecting two dots show repeated measurements (pre- and post- intoxication) from surviving mice in the final two groups. The two circles indicate the VNA levels from the three mice that did not survive BoNT/A1 intoxication. Dots within the shaded area represent VNA serum levels below detection.

In study 2, multiple groups of mice received Ad/VNA-BoNTA or Ad/VNA-control as in study 1, but in this study the duration between pre-treatments and toxin challenges was spread further and the study was extended to four months. In addition, serum was collected from all mice a week prior to toxin challenge. As in study 1, serum was collected from all survivors a week post-challenge. The percent survival data (Figure 4) shows that, as in study 1, all groups of mice pre-treated with control Ad/VNA-control failed to survive subsequent BoNT/A1 challenge. In contrast, all mice pre-treated with Ad/VNA-BoNTA, and challenged with toxin within 9 weeks, survived toxin challenge. In groups of Ad/VNA-BoNTA treated mice that were challenged with toxin either 13 or 17 weeks post-treatment, 2 of 5 mice survived (40%). Serum levels of VNA-BoNTA in all mice from long-term study 2 are shown in Figure 5. As with the study 1, serum levels varied widely within groups. This serum level was relatively stable within individual mice sampled during the two week interval. Serum Ad/VNA-BoNTA levels in all mice sampled beyond 10 weeks were <10 µg/ml, suggesting that expression of the VNA from the transgene diminishes after several months, consistent with the survival data from both studies.

As expected, all six Ad/VNA-BoNTA-treated mice that failed to survive the toxin challenge at 13 or 17 weeks post-treatment (study 2) had undetectable serum levels of VNA-BoNTA. As in the first study, some Ad/VNA-BoNTA-treated mice survived toxin challenge despite VNA-BoNTA levels below the 5 ng/ml detection limit. This is perhaps not surprising as we found shown in Figure 1B that VNA-BoNTA doses as low as 100–200 ng protected mice from similar BoNT/A1 challenges.

**Figure 6:**
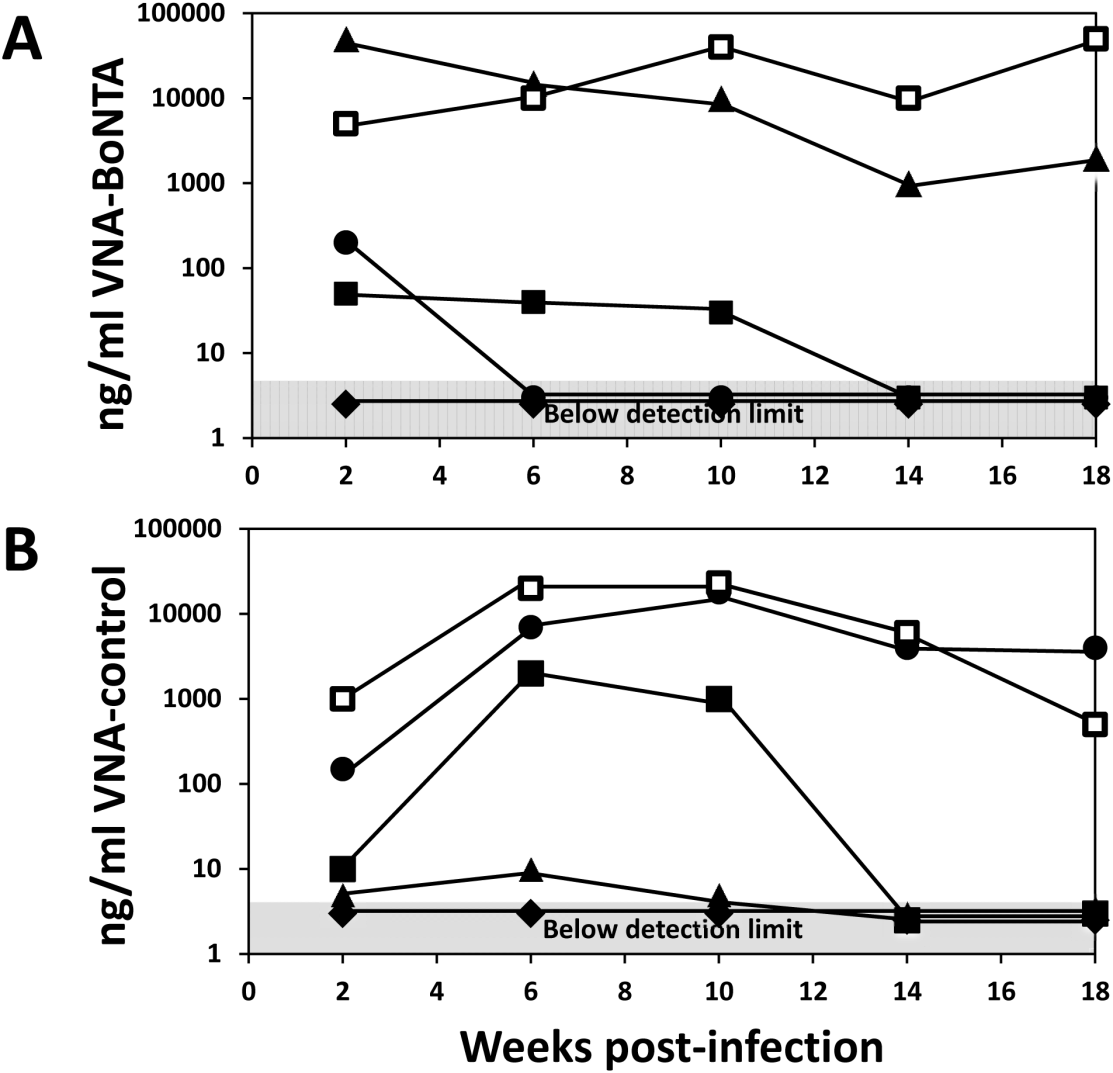
Persistence of serum VNAs. Groups of five mice (10 mice in total) were treated with 3×10^10^ vp of Ad/VNA-BoNTA (**A**) or Ad/VNA-control (**B**) on day 0 and bled two weeks later and then monthly for four months. The VNA concentrations (ng/ml) over time for individual mice are represented by the different symbols. Symbols within the shaded area represent VNA serum levels below detection.

Five mice treated with Ad/VNA-BoNTA or Ad/VNA-control were monitored monthly for serum VNA levels and the results are shown in Figure 6. Two mice in both groups maintained high VNA levels throughout the 18 week study period. One mouse in both groups never achieved detectable VNA levels. The four remaining mice all produced VNA during the first month or longer, but eventually the serum levels dropped to undetectable levels. VNA serum levels could diminish due to the loss of the transgene or the elimination of cells producing the VNA. Another possibility, explored below, was that VNA levels became undetectable due to the development of an antibody response that blocked their target binding and thus their detection by capture ELISA.

### Immunogenicity of VNAs expressed from a transgene

**Figure 7:**
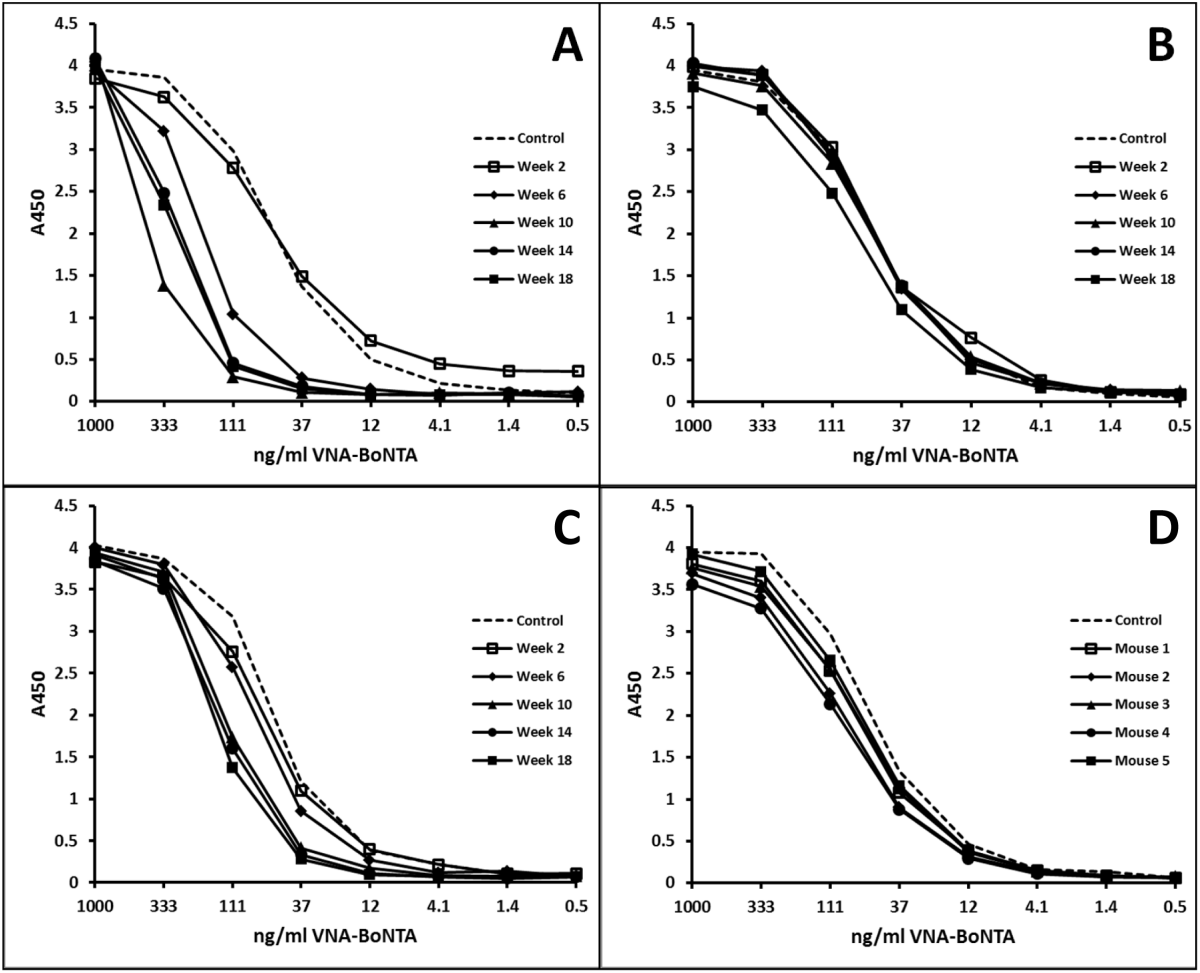
Some mice treated with Ad/VNA-BoNTA develop blocking antibodies. Serum from mice treated with Ad/VNA-BoNTA or Ad/VNA-control was collected monthly from weeks 2 through 18 post-treatment and VNA levels were assessed (data shown in Figure 6). **A–C.** Development of blocking Abs was investigated in the three Ad/VNA-BoNTA mice with low serum VNA levels (Figure 6A; square, circle and diamond symbols). The presence of blocking Abs was assayed by measuring the potency of mouse serum, 1∶100, to inhibit the binding of serially diluted recombinant VNA-BoNTA (Trx/H7/B5/ABP) to BoNT/A1. The presence of blocking Abs shifts the curve to the left. Each box represents sera collected from the same mice at the indicated times post-treatment with Ad/VNA-BoNTA. **D.** VNA-BoNTA blocking assays were performed as above in A–C employing serum from mice 18 weeks following treatment with Ad/VNA-control to detect VNA cross-specific blocking Abs elicited by expression of the control VNA. The serum source was the terminal bleed of the five mice in Figure 6B.

Prolonged VNA expression from the Ad/VNA transgenes could generate endogenous blocking anti-VNA antibodies that reduce antitoxin efficacy. An ELISA was developed to determine whether blocking Abs might contribute to lower VNA levels and reduce VNA-BoNTA efficacy. Mouse serum at 1∶100 was added to wells containing serially-diluted VNA-BoNTA protein and binding to BoNT/A was assessed. Therefore, the presence of blocking Abs interferes with VNA-toxin binding and shifts the curve to the left. Only sera with low endogenous VNA were tested since sera measured to have high endogenous VNA levels (using the BoNT/A binding assay) must contain VNA in excess of blocking Ab, thus obscuring detection. Figures 7A, B and C show blocking Ab results from the three mice bled monthly showing low VNA serum titers in Figure 6A (circle, square and diamond symbols). The mouse shown in Figure 7A developed blocking Abs between weeks 2 and 6 corresponding with a sharp drop in serum VNA levels (Figure 6A, circles). A second mouse did not develop blocking Abs (Figure 7B) despite maintaining VNA serum levels near 50 ng/ml for 10 weeks (Figure 6A, squares), which suggests that blocking Abs were not responsible for the loss of VNA serum expression at later times. The third mouse (Figure 7C) developed a weak blocking Ab response despite never developing detectable serum VNA-BoNTA titers (Figure 6A, diamonds). Figure 7D represents serum collected from five mice 18 weeks following treatment with Ad/VNA-control. Four of these mice expressed measurable VNA-control and yet none displayed detectable blocking Ab for VNA-BoNTA, suggesting prolonged serum expression of one VNA did not induce blocking Ab titers against a different VNA.

Assays such as shown in Figure 7 permit quantification of the blocking Ab titer in terms of the amount of VNA that can be blocked from target binding. The highest blocking Ab titer was found in the week 10 serum assayed in Figure 7A. At a 1∶100 dilution, this serum fully blocked 50 ng/ml VNA-BoNTA from binding to BoNT/A1, indicating that undiluted serum possessed a blocking Ab titer capable of neutralizing ∼5 µg/ml of VNA-BoNTA.

Similar blocking Ab assays were performed on serum from all 13 mice treated with Ad/VNA-BoNTA in the second long term study (Figure 5) in which serum contained <100 ng/ml endogenous VNA-BoNTA, including all six mice that did not survive BoNT/A challenge (weeks 12 and 16). None of the mice sampled in the first 6 weeks had developed detectable blocking Ab titers at euthanasia. Several of the mice tested 10 weeks or later post-treatment showed evidence of blocking Abs, including most of the mice that failed to survive BoNT/A1 challenge. The blocking Ab titers were always low, capable of neutralizing <2 µg/ml of VNA-BoNTA. Because only mice with low endogenous VNA-BoNTA could be assayed, we could not determine whether mice with high endogenous VNA-BoNTA levels also developed blocking Abs. However, if blocking Ab responses developed, they were insufficient to fully neutralize the VNA-BoNTA as all 63 mice with detectable VNA-BoNTA survived BoNT/A1 challenge in the two long term studies.

As shown in Figure 7, at least two mice treated with Ad/VNA-BoNTA maintained substantial blocking Ab titers for several months. Mice with both serum VNA and anti-VNA Abs may be at risk for Ag/Ab immune complex deposition, which can cause kidney damage by distorting renal glomeruli. Importantly, none of the mice in any of our studies had signs of kidney damage. Furthermore, the kidneys from some mice including those with known blocking Abs, were collected at 18 weeks after Ad/VNA-BoNTA injection and examined by a board certified veterinary pathologist. None of the glomeruli from these 10 mice (Figure 6) had glomerular basement membrane thickening or mesangial thickening evident by routine light microscopic examination. This indicates that Ag/Ab complexes were not deposited in sufficient amounts to cause structural damage as assessed by light microscopy.

### Ad/VNA-BoNTA protects mice from BoNT/A1 challenge when administered post-intoxication

**Figure 8:**
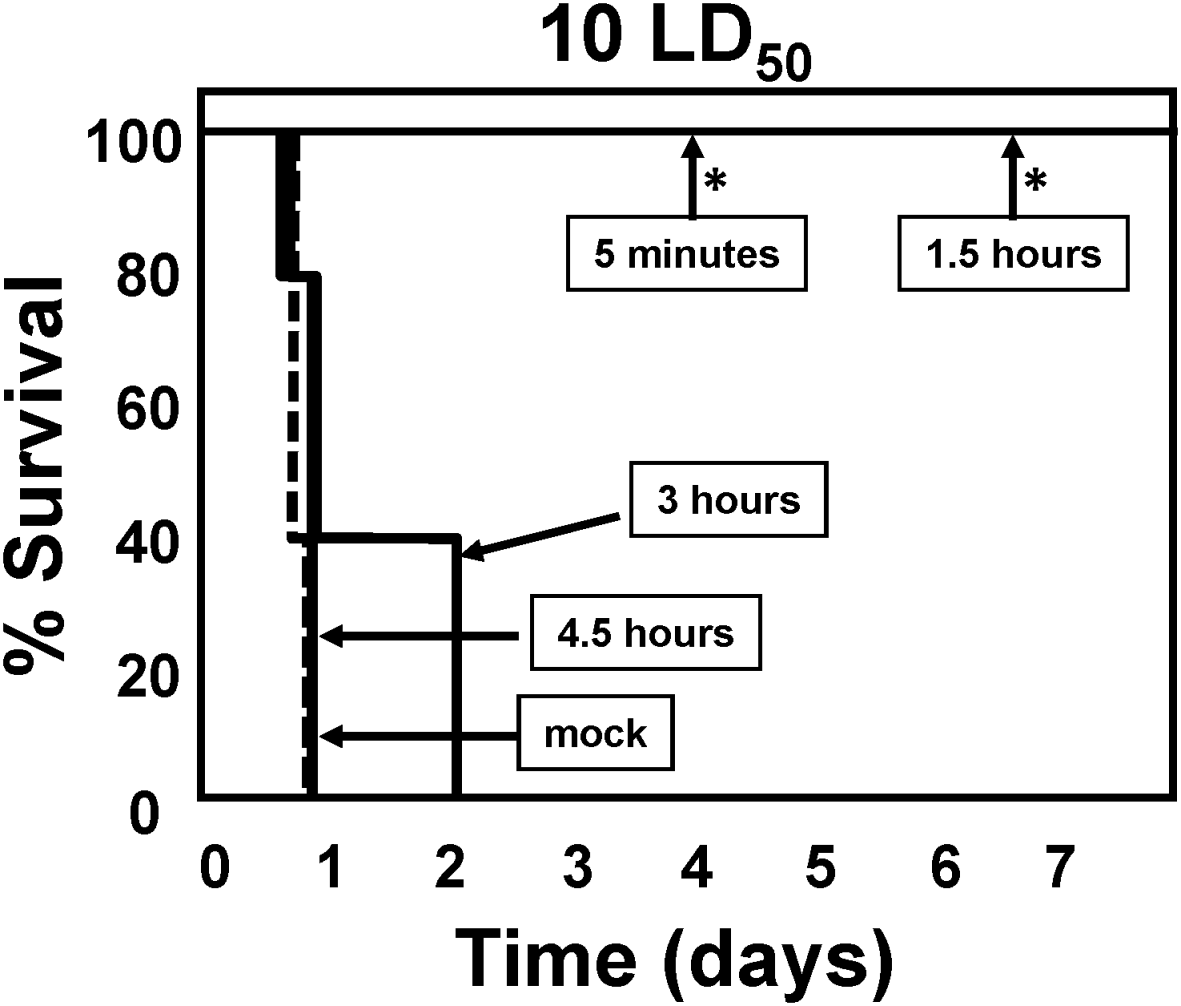
BoNT/A1 lethality in mice is prevented by rapid post-exposure treatment with Ad/VNA-BoNTA. A 10 LD_50_ dose of BoNT/A1 was administered by intraperitoneal injection to groups of five mice. Mice were then administered 3×10^10^ vp of Ad/VNA-BoNTA by intravenous injection at 5 minutes, 1.5, 3 or 4.5 hours later. A control group was given a mock treatment (PBS alone) 5 minutes following intoxication (dashed line). Symptoms of BoNT/A intoxication and survival were monitored and are shown as a survival curve for each group. The mock treated group is represented by a dashed line. An asterisk indicates a significant difference (p<0.05) from the control group. Surviving mice all showed moderate to severe abdominal breathing symptoms of botulism before recovering. In a prior study using the same doses, mice treated immediately following BoNT/A challenge with Ad/VNA-BoNTA, unlike those treated with Ad/VNA-control (0% survival after 24 hours), were fully protected and did not display any symptoms of botulism.

Mice exposed to 10 LD_50_ BoNT/A1 typically die from botulism within a day. These mice can be rescued by treatments with anti-toxin serum or with VNA-BoNTA during a window of about 3 hours [5]. To determine whether treatment with Ad/VNA-BoNTA could rescue mice exposed to 10 LD_50_ BoNT/A1, mice were first exposed to the toxin and then Ad/VNA-BoNTA was administered intravenously at various times post-intoxication. As shown in Figure 8, all mice survived when treated within 1.5 hours of intoxication (with symptoms of intoxication), while no mice survived when treated at 3 or 4.5 hours post-intoxication. Two additional groups of mice were treated with 100 LD_50_ BoNT/A1 and treated immediately with either Ad/VNA-BoNTA or 2 µg of recombinant VNA-BoNTA. All mice treated with Ad/VNA-BoNTA died within 5 hours while mice treated with antitoxin VNA-BoNTA protein survived without symptoms (not shown). These results show that a protective level of VNA-BoNTA was rapidly achieved within a few hours of Ad/BoNTA treatment, but that direct administration of antitoxin VNA-BoNTA protein is more effective in post-exposure situations.

## Discussion

Antibody therapy is used to treat many diseases, but its utility can be limited by manufacturing costs, shelf-life, and the health care infrastructure necessary for repeated parenteral administrations. We have developed an alternative therapeutic platform employing VNAs which can provide equivalent or superior antitoxin efficacy in animal models [5]–[7]. Passive antitoxin immunotherapy requires sustained antitoxin levels until the toxin is removed from the body and the threat of more exposure has ended. Here, we combine two different technologies that improve the persistence and levels of VNA antitoxins and test these using a mouse botulism model. First, an albumin binding peptide (ABP) was fused to the antitoxin VNA which significantly prolonged serum persistence. Second, we used an adenoviral gene transfer vector to deliver the VNA/ABP and promote prolonged serum expression of the antitoxin agent. In combination, we show that a single treatment with an Ad vector containing the VNA/ABP transgene (Ad/VNA) led to several months of protection from lethal BoNT/A1 exposure. Finally, we assessed the immunogenicity of VNAs and showed that, while some mice developing blocking Abs, this did not markedly impair efficacy or cause renal glomerular damage due to immune complex deposition.

Genetic delivery is a proven means to induce *in vivo* expression of antibodies for a variety of therapeutic applications, and offers an alternative to the standard practice of administering preparations of purified antibodies. The technique uses viral vectors such as baculovirus [21], rhabdovirus [22], Ad [9]–[13], and AAV [14]–[17] for gene transfer of mAb coding sequences and has been effective against both cancers and infectious diseases in *in vivo* experimental settings [9]–[11], [13], [14], [16], [23]. Recent work in our group and others has highlighted the potential to achieve targeted *in vivo* gene delivery [24], [25]. Further benefits should accrue as antibody agents can be targeted to body sites and portals of entry that are specifically relevant for toxin or infectious exposures; such as the upper and lower airways, gastrointestinal tract, or urogenital tract.

Gene therapy provides a means to achieve sustained antitoxin protection against diseases caused by bacterial toxins for both biodefense and infectious disease therapeutics. Other groups have previously employed Ad and AAV vectors to provide prolonged prophylactic protection against anthrax toxin [10], [11] by promoting expression of antitoxin mAbs or single-chain F_V_s (scFvs). In these studies, iv administration of Ad vector encoding an anti-anthrax scFv to mice, scFv was detected in serum over a 2-week period conferring antitoxin protection from 1 to 14 days prior to toxin challenge [11]. Similarly, the use of an iv treatment with Ad vector encoding anti-anthrax mAb provided protection against challenge after 1 day and continued through 8 weeks following administration [10]. Intrapleural administration of an AAV vector encoding the anti-anthrax mAb protected mice against toxin challenge from 2 weeks through a 6 month period [10], demonstrating delayed but sustained efficacy of AAV-delivered antitoxins.

An ideal antitoxin therapeutic should provide protection with an easily administered single application that is rapid-acting and also long-lasting, providing a high level of protection for the duration of a toxin exposure threat. The rapid transgene expression kinetics with Ad vectors renders them particularly applicable as antitoxin delivery vehicles. In the current study we used a single intravenous injection of an Ad vector to achieve very rapid protection from BoNT/A1 challenge (efficacy when administered 1.5 hrs post-exposure- Fig. 8) as well as long term protection (efficacy when administered two months prior to challenge- Fig. 4). These results support the potential of Ad/VNA agents as antitoxin therapeutics.

The use of antitoxin VNAs in gene transfer vectors offers clear advantages over conventional antitoxin mAbs, and some possible disadvantages, compared to conventional antitoxin mAbs. The advantages of VNAs are multiple, including: 1) easy manipulation to link toxin-neutralizing VHHs for increased potency and to target multiple toxins [5]–[7]; 2) VHH components can be obtained from pre-existing non-immune libraries [26], [27] and affinity-matured [28] to rapidly develop VHHs against new threats, and; 3) VHH-based agents are simpler to produce and store due to their unusual stability and favorable expression properties (reviewed by [29], [30]). A potential limitation is heightened immunogenicity compared to human mAbs due to their foreign origin. Although VHHs have been described as non-immunogenic [29], [30], our results demonstrate that anti-VHH blocking antibodies can develop following prolonged exposure. However, it is not known how these anti-VHH responses compare to the anti-idiotypic responses that follow prolonged exposure to mAbs [31]. VNA immunogenicity can be minimized by humanization of the VHHs [32] or promoting immune tolerance as a consequence of the high transgene expression [33], [34]. It is worth noting that eliciting anti-VNA antibodies that don’t block neutralization could improve efficacy by promoting Fc effector functions such as toxin clearance [5].

The broad range in VNA serum levels we found in different mice treated with the same dose of Ad/VNA has been observed previously [12] yet remains unexplained. One possibility is that hepatic Kupffer cells sequester iv-delivered virus, leading to nonlinear Ad liver transduction [35]–[41]. Here, we show that the VNA levels in the first four days following Ad/VNA iv administration were consistently greater than 10 µg/ml (Tables 1 and 2), an observation consistent with several other studies not shown. After four days, our findings indicate that VNA serum levels can change substantially, sometimes remaining very high for several months but often dropping precipitously in the days or weeks that follow. The reasons for a decrease in VNA production may include the transcriptional shutoff of the CMV expression cassette, turnover of transduced cells, and immune responses against VNAs as well as the VNA-producing cells, as has been documented in a variety of comparable experimental settings [42]–[44]. The properties of Ad/VNA vectors need to be further studied with the goal to develop improved administration methods or improved Ad vectors with which less variable and more persistent VNA serum levels are achieved.

Fusion of the VNA-BoNTA to an ABP provided a substantial improvement in the serum stability of the VNA in mice, changing the serum T_1/2_ from about 1–2 hours to between 1–2 days based on the persistence of protection from BoNT/A1 challenge that could be achieved following administration of the purified protein. While improved pharmacokinetic properties may be less important in gene therapy applications where the VNA is continuously expressed, the improved serum persistence should substantially improve the steady-state serum levels of the VNA which may be important in some applications. The ABP that we employed was selected for its binding properties to mouse albumin and we found that its affinity for albumin from other animal sources varied widely. As treatments are initiated in other animals or humans, it may be necessary to identify ABPs with improved affinity for the albumin in that serum. Many other alternative strategies are also becoming available for improving the serum persistence of protein therapeutics [45].

Previous studies employing VNA antitoxins for BoNT/A, BoNT/B, ricin and Shiga toxins [5]–[7] have shown that co-administration of an anti-tag effector Ab (efAb) that binds to multiple tags built into the VNA leads to substantial improvements in the in vivo antitoxin efficacy of treatments, particularly for high dose toxin challenges. Here we confirm prior results [5] showing that efAb is not necessary for full protective efficacy with low toxin challenge doses (10 LD_50_) and find that VNA expression levels in serum as low as ∼5 ng/ml were sufficient. Since most Ad/VNA-treated mice achieved much higher VNA serum levels, it may be that most mice would be protected from higher dose toxin challenges. If increased antitoxin potency is required in other models of intoxication, it should be possible to engineer the Ad vehicle to co-express an efAb to boost the VNA potency.

Here, our studies show that a single dose of Ad/VNA-BoNTA provides protection from BoNT/A1 intoxication within about an hour and this protection lasts for several months. We envision that this general strategy has a broad range of beneficial applications including protecting military personnel from possible future exposure to bioweapons, and as prophylactic or post-exposure treatment of patients with toxicoinfectious diseases such as *Clostridium difficile* or pathogenic *Escherichia coli*.

## Methods and Materials

### Ethics statement

All studies followed the Guide for the Care and Use of Laboratory Animals of the National Institutes of Health and were approved by the Tufts University Institutional Animal Care and Use Committee (IACUC) under Protocols #G2010-60 and G874-07.

### Toxins

BoNT/A1 complex (Metabiologics Inc.) was used for in vivo assays. Each batch was evaluated to determine the LD_50_ dose. All procedures utilizing BoNT/A1 were performed within a CDC-registered Select Agent laboratory.

### Adenovirus vector construction and preparation

The generation of recombinant replication-incompetent Ad5-based vectors was carried out essentially as described elsewhere [46]. Briefly, genome of Ad/VNA-BoNTA vector was generated using pShCMV-JGF7 shuttle plasmid. The plasmid pShCMV-JGF7 was constructed by subcloning the DNA encoding VNA-BoNT/A (Fig. S2), a VHH heterodimer recognizing BoNT/A1 (ciA-H7/B5 [5]) with a carboxyl terminal ABP, to create a transcription unit under control of the strong mammalian CMV promoter within plasmid pAdTrack [47], replacing GFP, and retaining the bovine growth hormone polyadenylation signal. The genome of Ad/VNA-Stx vector was similarly generated using pShCMV-JHQ9 shuttle plasmid constructed by subcloning DNA encoding VNA-Stx (Fig. S2), a VHH heterotrimer (A9/A5/G1 [6]) recognizing both Stx1 and Stx2. Ad/VNA-control genome was constructed as Ad/VNA-BoNTA by preparing pShCMV-control containing DNA encoding a VHH heterodimer consisting of two VHHs recognizing *C. difficile* toxins. Both pShCMV-JGF7, pShCMV-JHQ9 and pShCMV-control shuttle plasmids were linearized with *BstZ17*I and employed for homologous recombination with pAdEasy-1 plasmid [47] using *E*. coli BJ5183-AD-1 cells as recommended by the manufacturer (Agilent Technologies, Inc., Santa Clara, CA) to generate pAd/VNA-BoNTA and pAd/VNA-Stx rescue plasmid, respectively. The resultant pAd/VNA-BoNTA, pAd/VNA-Stx and pAd/VNA-control plasmids containing viral genomes were validated by PCR, restriction analysis, and partial sequencing, and then were linearized with *Pac*I to release the inverted terminal repeats of the viral genomic DNA and transfected into 293 cells [48] to rescue replication-incompetent Ad/VNA-BoNTA, Ad/VNA-Stx or Ad/VNA-control vectors. The newly rescued Ad vectors were propagated on 911 cells [49], purified by centrifugation on CsCl gradients according to standard protocol, and dialyzed against phosphate-buffered saline (PBS) (8 mM Na_2_HPO_4_, 2 mM KH_2_PO_4_ [pH 7.4], 137 mM NaCl, 2.7 mM KCl) containing 10% glycerol. The titers of physical viral particles (vp) were determined by the methods of Maizel et al. [50].

### Standard mouse toxin lethality assay

Adenovirus vectors were administered prior to or following BoNT/A1 intoxication and their efficacies in protecting mice from botulism were evaluated using BoNT/A1 lethality assays [5]. All procedures were performed under approved Institutional Animal Care and Use protocols. The lethality assays utilized adult, ∼20 g female CD-1 mice (Charles River Labs) housed in standard shoebox cages at 5 mice/cage. Following arrival, mice were observed at least twice daily and cages containing corncob bedding and nestlets along with standard rodent chow within the cage rack and water ad libitum were changed twice weekly. Throughout each study, mice were also offered food on the cage bottom along with both nutritional (Napa Nectar; Lenderking #88-0001) and hydration (Boost; Clear H2O #72-04-5022) supplement gels. One day prior to initiation of each study, mice were weighed and sorted to reduce intergroup (n = 5) weight variation. Following administration of BoNT/A1, mice were observed a minimum of four times each day between 9 a.m. and midnight with additional checks throughout peak periods of mortality in an effort to ensure accurate documentation of time to death data. Time to death was defined as the time at which a mouse was found dead or was euthanized due to severe morbidity. At each check, a standard score sheet was utilized to document the overall disposition and clinical symptoms exhibited by each mouse. Mice which were observed to be open-mouth breathing, lethargic, unresponsive or moribund were euthanized via carbon dioxide asphyxiation followed by cervical dislocation. Adenovirus vectors were administered iv into the lateral tail vain (200 µl), im into the thigh (50 µl), ip into the abdomen (200 µl) or sq over the dorsum (200 µl). Ten LD_50_ BoNT/A1 was administered iv into the lateral tail vein at various times, prior to or following treatment. Blood was obtained prior to BoNT/A1 challenge and from survivors following isoflurane anesthesia from the retro-orbital sinus. Following isolation, serum was stored at −20°C prior to immunoassay.

### Capture ELISAs to measure serum VNA levels

Maxisorp C96 immunoplates (Nunc) or 96-well tissue culture plates (Corning) were coated overnight with 0.5 or 1 µg of ciBoNTA [5]. The standard for VNA-BoNTA was a recombinant thioredoxin-fusion protein (Trx/H7/B5/ABP), identical to VNA-BoNTA except that it was produced with an amino terminal thioredoxin fusion partner purified as previously described [5] This standard was ‘spiked’ into normal mouse serum at 80 nM. ELISAs were performed in which spiked serum or test sera were applied to a well at a 1∶10 dilution followed by seven 1∶5 serial dilutions. VNA bound to the ciBoNTA capture reagent was detected with HRP-anti-E-tag mAb (GE Healthcare). VNA levels in test sera were estimated by comparison to spiked serum that was included in every ELISA plate. This level was a subjective estimate based on EC_50_ values [6] relative to the standard. Because plots sometimes varied slightly in shape or peak signal, and low concentration samples did not produce a peak signal so as to permit EC_50_ to be unambiguously determined, all values must be considered approximate and interpreted as such. Samples assayed in multiple different assays always produced results varying less than five-fold. Most serum samples, and all samples estimated to be less than ∼5 nM, were assayed at least twice. Typically, signals two-fold above background were achieved in dilutions of VNA-BoNTA standard down to about 150 pM (5 ng/ml). Thus, all assays measuring 150 pM or below in multiple separate assays were considered as ‘below detection limit’.

VNA-Stx serum level assays were performed as for VNA-BoNTA with the following differences. All assays employed Nunc Immunoplates. The internal standard used to estimate VNA-Stx levels was a recombinant thioredoxin fusion protein otherwise identical to VNA-Stx (Trx/A9/A5/G1/ABP [6]). Plates were coated with 1 µg of Stx2 (Phoenix Lab, Tufts University, Boston, MA). Detection limits of spiked mouse sera were about 30 pM.

### Blocking Ab assay

Some mice receiving Ad/VNA treatments were assayed for the development of serum anti-VNA antibodies capable of blocking VNA binding to its target. Only serum containing endogenous VNA levels below about 100 ng/ml could be effectively assayed. A dilution ELISA was performed in which a VNA standard was assayed for binding to target in the presence test mouse serum or control mouse serum. 96 well plates were coated with ciBoNTA as described above and columns pre-treated with a 1∶100 dilution of sera from individual mice at varying times post-treatment with Ad/VNA or untreated controls. A 1∶3 dilution series was prepared down the column, beginning with 1 µg of Trx/H7/B5/ABP (containing 1∶100 dilution of the same mouse serum). VNA binding to target was measured and the EC_50_ determined as above. Mouse sera that caused more than a two-fold shift in EC_50_ compared to untreated control sera were considered to contain measurable blocking Abs.

### Statistical analysis

Survival data was analyzed using SigmaPlot (Version 12.3, Systat Software, Inc.). Percent survival of treated vs. control mice was analyzed using an unpaired t-test followed by a Mann-Whitney rank sum test. Prolongation of survival of treated vs. control mice was evaluated via Kaplan-Meier log-rank analysis.

## Supporting Information

Figure S1 **ABP binding affinity and specificity.** Dilution ELISAs were performed to assess the binding of VNA-BoNTA to various mammalian sera. ELISA plates were coated with 1∶1000 dilutions of serum from mouse, rat, rabbit, pig, human and bovine sources. A 1∶3 dilution series beginning with a 330 nM solution of VNA-H7/B5 (dashed line, squares) or VNA-H7/B5/ABP (solid line, circles) was assayed for binding to the different sera. Several additional ELISAs were done with purified mouse, human and pig albumin and produced similar outcomes.
(TIF)

Figure S2 **Ad/VNA-BoNTA and Ad/VNA-Stx transcription units. (A)** Diagram of the transcription unit engineered to promote expression and secretion of VNA-BoNTA in cells transduced by Ad/VNA-BoNTA. DNA encoding VNA-BoNTA was inserted between a CMV promoter and the SV40 polyadenylation (polyA) site in frame with DNA encoding a signal peptide (SP) from the mouse Ig kappa-chain gene (see Methods and Materials). VNA-BoNTA (H7/B5/ABP) contains two BoNT/A1-neutralizing VHHs, ciA-H7 and ciA-B5 [5] joined by DNA encoding a 15 amino acid flexible spacer, (GGGGS)_3_ (black box). DNA encoding the 14 amino acid albumin-binding-peptide (ABP), DICLPRWGCLEWED [20] is fused in frame to the carboxyl end of the H7/B5 coding DNA, separated by a GGGGS spacer. Two copies of the E-tag peptide epitope are also encoded (E), one at the amino end of ciA-H7 and the other between ciA-B5 and ABP, and used for detection in this study. Boxes are not to scale. **(B)** Amino acid sequence of the encoded VNA-BoNTA. The complete amino acid sequence of the encoded VNA-BoNTA within Ad/VNA-BoNTA as diagrammed in A is shown. For clarity, the eight different protein domains shown in A are separated by///. **(C)** Amino acid sequence of VNA-Stx (VNA-A9/A5/G1/ABP) encoded by Ad/VNA-Stx. The complete amino acid sequence VNA-Stx [6] within Ad/VNA-Stx is shown. For clarity, the different protein domains (SP; three Stx-neutralizing VHHs Stx1-A9, Stx-A5, Stx2-G1 [6]; E; ABP) are separated by///as in B.
(TIF)

Figure S3 **Serum VNA titers following Ad/VNA treatments via different routes of administration.** Dilution ELISAs were performed to assess the VNA-BoNTA **(A)** or VNA-Stx **(B)** titers from five individual mice (solid lines) four days following treatment with 3×10^10^ vp of Ad/VNA-BoNA or Ad/VNA-Stx respectively. Treatments were administered via intravenous, intraperitoneal, intramuscular or subcutaneous routes (separate boxes). ELISA plates were coated with 1 µg/ml of ciBoNT/A **(A)** or Stx2 **(B)**. The first well contained a 1∶10 dilution of serum followed by a 1∶5 dilution series. A control mouse serum (dashed lines) was spiked with 400 nM of recombinant VNA-BoNTA (Trx/H7/B5/ABP) **(A)**, or VNA-Stx (Trx/A9/A5/G1/ABP) **(B)**. VNA binding to toxin was detected with HRP/anti-E-tag (vertical axis). The horizontal axis label shows the concentration of the Trx/VNA standard.
(TIFF)
