# Supplementary figures and images for: Prolonged Prophylactic Protection from Botulism with a Single Adenovirus Treatment Promoting Serum Expression of a VHH-Based Antitoxin Protein
Source: PLoS One. 2014 Aug 29;9(8):e106422. doi: 10.1371/journal.pone.0106422 (PMC4149568; doi:10.1371/journal.pone.0106422)

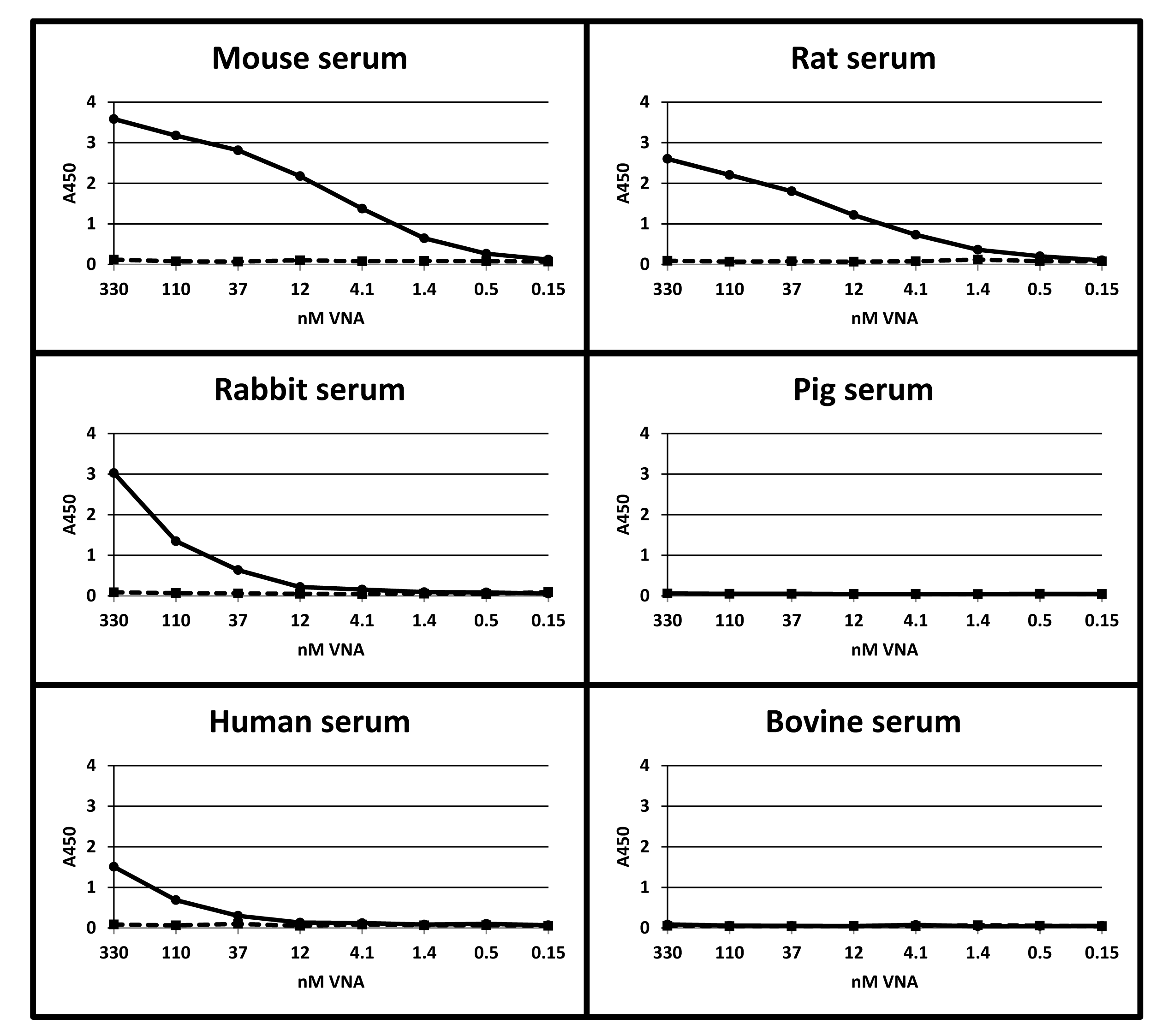

Supplement: Figure S1 — ABP binding affinity and specificity. Dilution ELISAs were performed to assess the binding of VNA-BoNTA to various mammalian sera. ELISA plates were coated with 1∶1000 dilutions of serum from mouse, rat, rabbit, pig, human and bovine sources. A 1∶3 dilution series beginning with a 330 nM solution of VNA-H7/B5 (dashed line, squares) or VNA-H7/B5/ABP (solid line, circles) was assayed for binding to the different sera. Several additional ELISAs were done with purified mouse, human and pig albumin and produced similar outcomes. (TIF) [file pone.0106422.s001.tif]

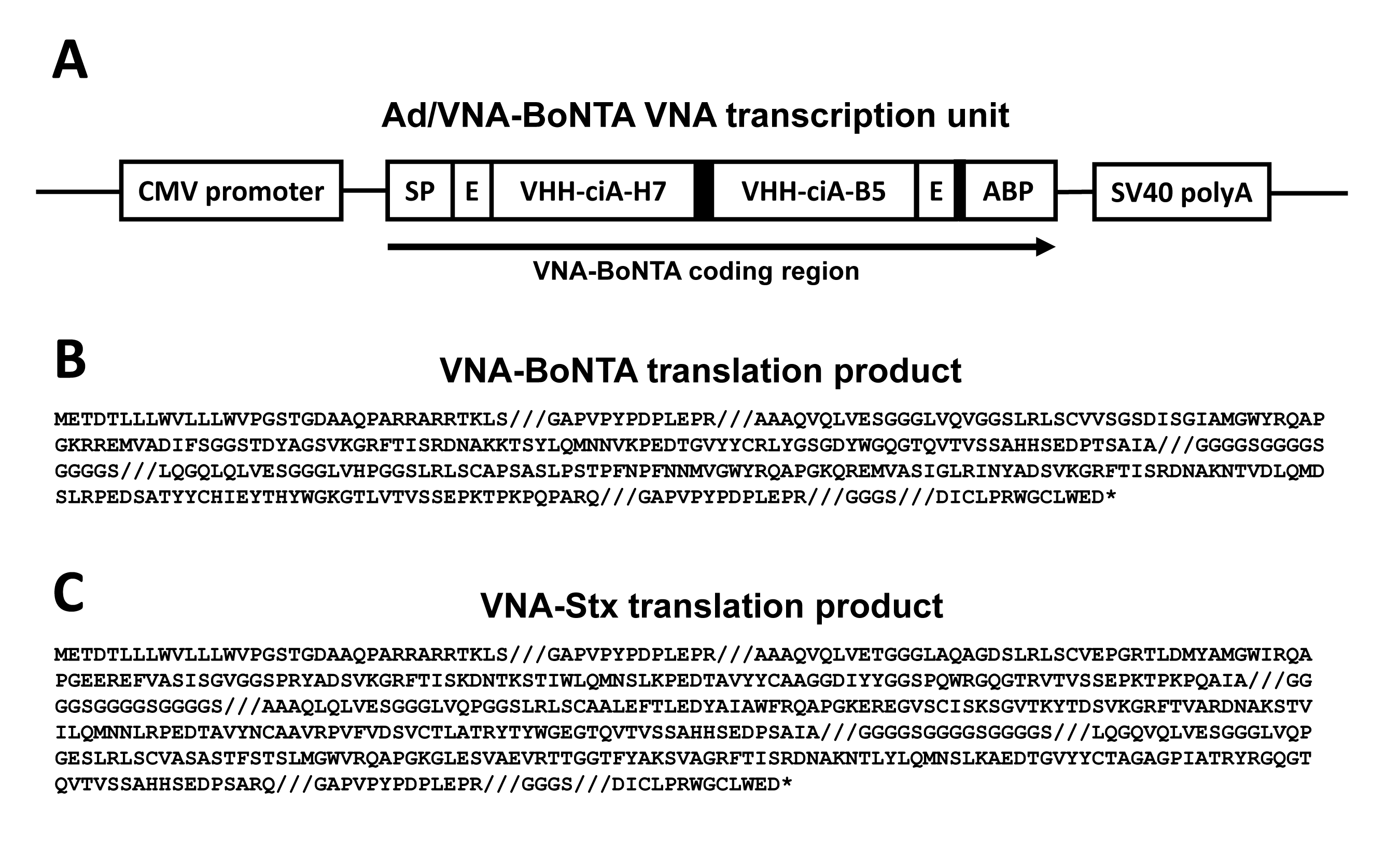

Supplement: Figure S2 — Ad/VNA-BoNTA and Ad/VNA-Stx transcription units. (A) Diagram of the transcription unit engineered to promote expression and secretion of VNA-BoNTA in cells transduced by Ad/VNA-BoNTA. DNA encoding VNA-BoNTA was inserted between a CMV promoter and the SV40 polyadenylation (polyA) site in frame with DNA encoding a signal peptide (SP) from the mouse Ig kappa-chain gene (see Methods and Materials). VNA-BoNTA (H7/B5/ABP) contains two BoNT/A1-neutralizing VHHs, ciA-H7 and ciA-B5 [5] joined by DNA encoding a 15 amino acid flexible spacer, (GGGGS)3 (black box). DNA encoding the 14 amino acid albumin-binding-peptide (ABP), DICLPRWGCLEWED [20] is fused in frame to the carboxyl end of the H7/B5 coding DNA, separated by a GGGGS spacer. Two copies of the E-tag peptide epitope are also encoded (E), one at the amino end of ciA-H7 and the other between ciA-B5 and ABP, and used for detection in this study. Boxes are not to scale. (B) Amino acid sequence of the encoded VNA-BoNTA. The complete amino acid sequence of the encoded VNA-BoNTA within Ad/VNA-BoNTA as diagrammed in A is shown. For clarity, the eight different protein domains shown in A are separated by///. (C) Amino acid sequence of VNA-Stx (VNA-A9/A5/G1/ABP) encoded by Ad/VNA-Stx. The complete amino acid sequence VNA-Stx [6] within Ad/VNA-Stx is shown. For clarity, the different protein domains (SP; three Stx-neutralizing VHHs Stx1-A9, Stx-A5, Stx2-G1 [6]; E; ABP) are separated by///as in B. (TIF) [file pone.0106422.s002.tif]

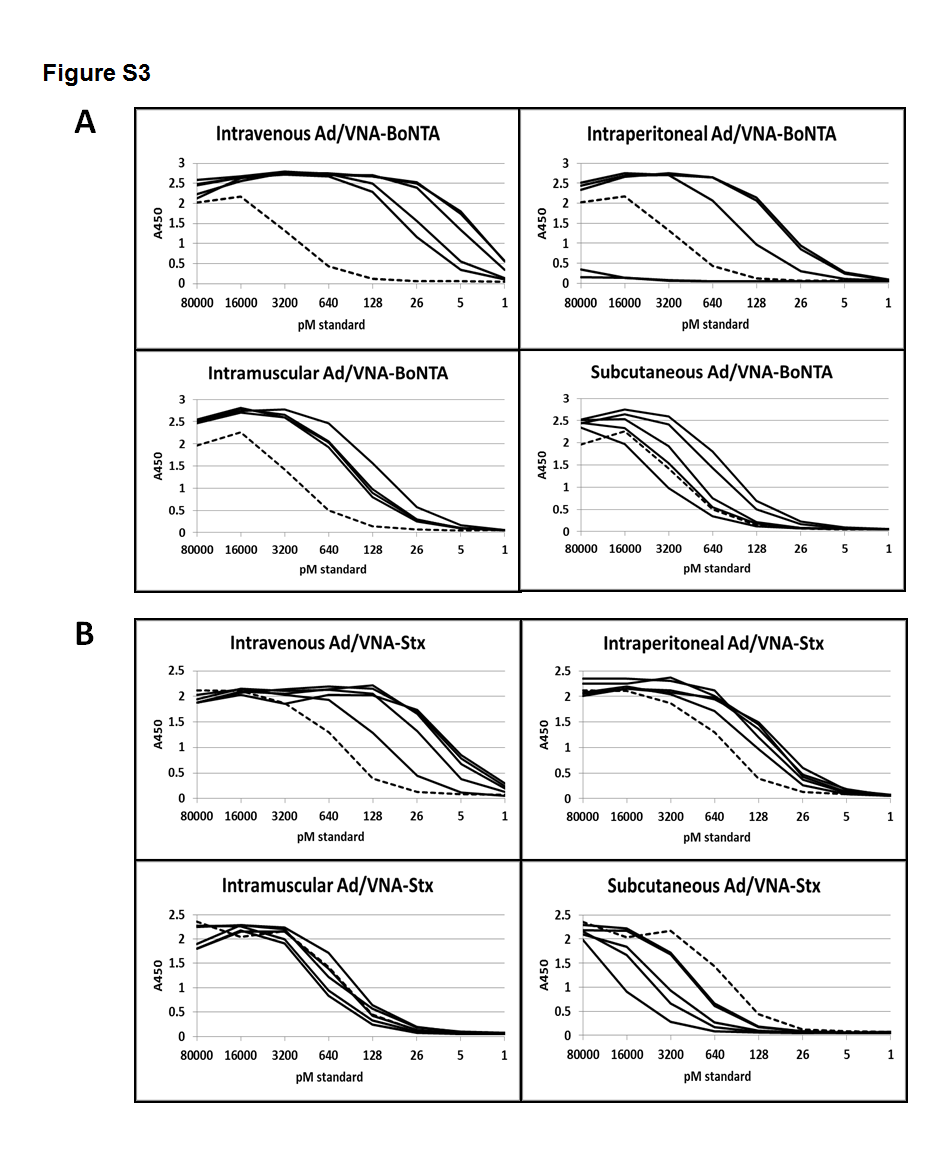

Supplement: Figure S3 — Serum VNA titers following Ad/VNA treatments via different routes of administration. Dilution ELISAs were performed to assess the VNA-BoNTA (A) or VNA-Stx (B) titers from five individual mice (solid lines) four days following treatment with 3×10^10^ vp of Ad/VNA-BoNA or Ad/VNA-Stx respectively. Treatments were administered via intravenous, intraperitoneal, intramuscular or subcutaneous routes (separate boxes). ELISA plates were coated with 1 µg/ml of ciBoNT/A (A) or Stx2 (B). The first well contained a 1∶10 dilution of serum followed by a 1∶5 dilution series. A control mouse serum (dashed lines) was spiked with 400 nM of recombinant VNA-BoNTA (Trx/H7/B5/ABP) (A), or VNA-Stx (Trx/A9/A5/G1/ABP) (B). VNA binding to toxin was detected with HRP/anti-E-tag (vertical axis). The horizontal axis label shows the concentration of the Trx/VNA standard. (TIFF) [file pone.0106422.s003.tiff]
